# Geo-located Twitter as proxy for global mobility patterns

**DOI:** 10.1080/15230406.2014.890072

**Published:** 2014-02-26

**Authors:** Bartosz Hawelka, Izabela Sitko, Euro Beinat, Stanislav Sobolevsky, Pavlos Kazakopoulos, Carlo Ratti

**Affiliations:** ^a^ Department of Geoinformatics – Z_GIS, GISscience Doctoral College, University of Salzburg, Salzburg, Austria; ^b^ SENSEable City Laboratory, Massachusetts Institute of Technology, Cambridge, MA, USA

**Keywords:** geo-located Twitter, global mobility patterns, community detection, collective sensing

## Abstract

Pervasive presence of location-sharing services made it possible for researchers to gain an unprecedented access to the direct records of human activity in space and time. This article analyses geo-located Twitter messages in order to uncover global patterns of human mobility. Based on a dataset of almost a billion tweets recorded in 2012, we estimate the volume of international travelers by country of residence. Mobility profiles of different nations were examined based on such characteristics as mobility rate, radius of gyration, diversity of destinations, and inflow–outflow balance. Temporal patterns disclose the universally valid seasons of increased international mobility and the particular character of international travels of different nations. Our analysis of the community structure of the Twitter mobility network reveals spatially cohesive regions that follow the regional division of the world. We validate our result using global tourism statistics and mobility models provided by other authors and argue that Twitter is exceptionally useful for understanding and quantifying global mobility patterns.

## Introduction

Reliable and effective monitoring of worldwide mobility patterns plays an important role in studies exploring migration flows (Castles and Miller 1998; Greenwood 1985; Sassen 1999) and tourist activity (Miguéns and Mendes 2008), as well as for examining the spread of diseases and for epidemic modeling (Bajardi et al. 2011; Balcan et al. 2009). Traditionally, those studies relied either on aggregated and temporally sparse official statistics or on selective, small-scale observations and surveys. In a more recent approach, worldwide mobility was approximated using air traffic volumes (Barrat et al. 2004). However, this dataset of potentially global coverage is biased toward just one mode of transportation and is, in many cases, difficult to obtain. Data about location-sharing services provide researchers with much more accurate records of human activity. Each day, millions of individuals leave behind digital traces of their activities by using mobile phones, credit cards, or social media. Most of those traces can be located in space and time, and thus they constitute a valuable source for human mobility studies.

Out of several types of collectively sensed data, cellular phone records have been the most intensively explored for analysis of human mobility. Because cell phones are almost universally used, data about their use have led to important findings about movement in urban (Calabrese et al. 2010; Kang et al. 2012), regional (Calabrese et al. 2013; Sagl et al. 2012), and national scales (Krings et al. 2009; Simini et al. 2012). High fragmentation of the mobile telecom market, however, precludes the availability of a worldwide dataset on cell phone use. In this case, social media data are a good alternative. Despite its lower penetration and a potential bias toward younger populations, social media’s popularity and representativeness are high and growing (Gesenhues 2013), and in most cases the media are global by design.

**Figure 1.**
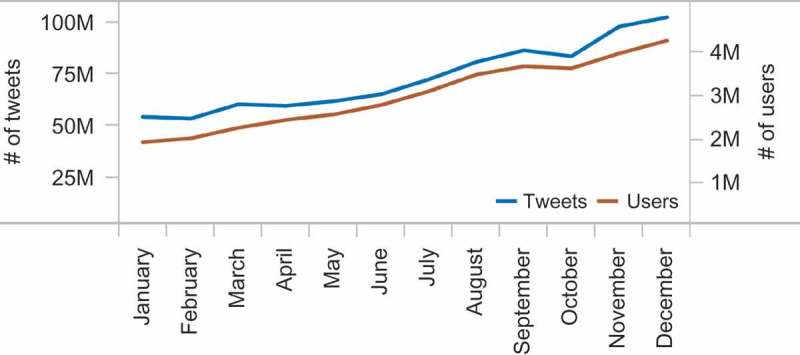
Number of geo-located tweets (blue line) and users (orange line) per month in 2012.

In this study, we attempt to uncover global mobility patterns and compare mobility characteristics of different nations. Our work is based on the data from Twitter – one of the most popular social media platforms, with over 500 million users registered by mid-2013 (Twitter Statistics 2013). Initially established in the USA, the service has quickly spread to other countries (Java et al. 2007; Leetaru et al. 2013; Mocanu et al. 2013), becoming a worldwide phenomenon. By design, Twitter is an open and public medium, which practically limits privacy consideration, especially in studies such as ours which examine collective rather than individual patterns of human behavior. In particular, we take advantage of tweets augmented with explicit geographic coordinates provided by either GPS embedded in a mobile device or the IP address of a computer. These geo-located tweets account for around 1% of the total feed (Morstatter et al. 2013). However, thanks to the increasing penetration of smart devices and mobile applications, the volume of the geo-located Twitter has been constantly growing (Figure 1), becoming an invaluable register of human traces in space and time. The absolute volume of 3.5M geo-located tweets per day (authors’ calculation for December 2012) appears as a promising base for carrying out a worldwide mobility analysis, which is the objective of our exploration.

Because of richness yet simplicity of the medium, Twitter has already been the subject of many studies for a variety of applications. First explorations focused on the properties of Twitter as a social network, proving its global character and scientific potential as early as one year after its launch (Huberman, Romero, and Wu 2008; Java et al. 2007; Kwak et al. 2010). Another line of research examined the content of tweets to assess society’s mood (Bollen, Pepe, and Mao 2011; Golder and Macy 2011; Pak and Paroubek 2010). Recently, this research was done also with a geographic perspective (Frank et al. 2013; Mitchell et al. 2013). Yet another area that has received much attention is crisis management (MacEachren et al. 2011; Sakaki, Okazaki, and Matsuo 2010; Thom et al. 2012), where the emphasis was on the detection of anomalous activity and the potential of a locally generated content to inform emergency services. Geo-located Twitter data have been used to inform urban management and planning (Frias-Martinez et al. 2012; Wakamiya, Lee, and Sumiya 2011), as well as in public health assessment (Ghosh and Guha 2013). All the aforementioned studies were spatially selective, focusing on specific study areas. The global perspective was introduced in the study of Kamath et al. (2012), with the analysis of the geographic spread of hashtags. Leetaru et al. (2013) attempted to describe the geography of Twitter based on a one-month sample of global geo-located tweets, while Mocanu et al. (2013) described the global distribution of the different languages used while tweeting.

Given the well-known role of location information in social networking services, attempts to convert this information into mobility characteristics remain relatively sparse. The most important foundation was provided by Cheng et al. (2011), who analyzed different aspects of mobility based on Twitter check-ins, which were at that point dominated by the feed from another location-sharing service – Foursquare. The study was extensive in scope but was limited by the availability of temporal data. There were other studies, such as that by Cho, Myers, and Leskovec (2011), who used data from the Gowalla and Brightkite services to model the influence of human mobility on social ties, or by Noulas et al. (2012) who focused on intra-urban mobility derived from Foursquare check-ins.

In this article, we present a global study of mobility based on the analysis of Twitter data and the mobility characteristics of different nations. We also seek to discover spatial patterns and clusters of regional mobility. Finally, we attempt to validate the representativeness of geo-located Twitter as a global source for mobility data. The article is organized as follows. First, we describe the dataset and illustrate a method to assign users to a country of residence, hence enabling the determination of home users and foreign visitors. Next, we present and compare mobility profiles of various countries, as well as the temporal patterns of inflow and outflow dynamics. Further, we explore country-to-country network of travels and delineate global regions of mobility. Finally, we validate the results in two ways: (i) through a comparison of the Twitter data with worldwide tourism statistics and (ii) with commonly used models of human mobility.

## Data pre-processing

Our study relies on one full year of geo-located tweets^1^ that were posted by users all over the world between January 1, 2012, and December 31, 2012. The database consists of 944M records generated by a total of 13M users. The stream was gathered through the Twitter Streaming API.^2^ Although the service sets a limit on how much data can be accessed to less than 1% of the total Twitter stream, the total geo-located content was found not to exceed this restriction (Morstatter et al. 2013). Therefore, we believe that we successfully collected a complete picture of global geo-located Twitter activity in 2012.

The database had to be cleaned before the analysis to prevent the contamination of mobility statistics by errors and artificial tweeting noise. First, we examined all the consecutive locations of a single user and excluded those that implied a user relocating with a speed over 1000 km/h, i.e., faster than a passenger plane. Further, we filtered out such activities on Twitter as web advertising (e.g., tweetmyjob), web gaming (e.g., map-game), or web reporting (e.g., sandaysoft). Those services can generate significant volumes of data, which do not reflect human physical presence in either the reported place or time. To correct for this artificial tweeting noise, we checked the popularity of a message’s source, assuming that those with only few users can probably be classified as an artificial activity. As the threshold, we used a cumulative popularity among 95% of users, constructing the ranking separately for each country. All tweeting sources falling below the threshold were discarded from further analysis. In total, the refinement procedure preserved 98% of users and 95% of tweets from the initial database.

## Definition of users’ countries of residence

An essential first step in our cross-country mobility analysis was an explicit assignment of each user to a country of residence. This made our work different from most of the other Twitter studies that usually did not attempt to uncover users’ origin and characterized a study area using only the total volume of tweets observed in this area (e.g., Mocanu et al. 2013). While for certain research problems this approach is suitable, from the perspective of a global mobility study, the differentiation between residents and visitors is crucial. It enables a clear definition of the origin and destination of travels and reveals which nation is traveling where and when. Taking advantage of the history of tweeting records of each user, we defined country of residence as the country where the user had issued most of the tweets. Once the country of residence was identified, the user’s activity in any other country of the world was considered as traveling behavior, and the user was counted as a visitor to that country.

We use the country definitions provided by the global administrative areas spatial database, which divides the world into 253 territories (Global Administrative Areas 2012). Twitter “residents” were identified in 243 of these territories, with the number of users greatly varying among different countries. The unquestionable leader is USA with over 3.8M users, followed by the UK, Indonesia, Brazil, Japan, and Spain with over 500K users each. There are also countries and territories with only few or no Twitter users assigned.

**Figure 2.**
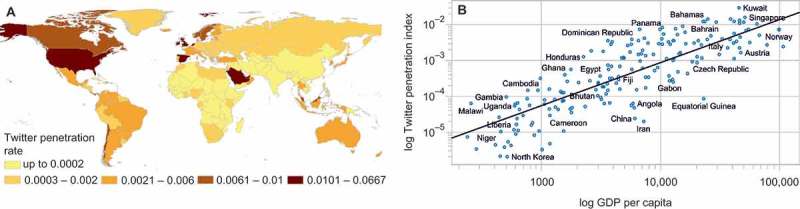
Twitter penetration rate across the countries of the world (A). Spatial distribution of the index. (B) Superlinear scaling of the penetration rate with per capita GDP of a country. *R* ^2^ coefficient equals 0.70.

To evaluate the representativeness of Twitter in a given area, we used a more illustrative metric, the penetration rate, defined as the ratio between the number of Twitter users and the population of a country. As expected, this rate does not distribute uniformly across the globe and scales superlinearly with the level of a country’s economic development (expressed as GDP per capita) (Figure 2A and B). Although this property has already been described by Mocanu et al. (2013) and others, we did observe that the goodness of a power law fit increased when considering penetration of only residents rather than all Twitter users appearing in a country. In the analysis, we exclude all countries with a penetration rate below 0.05% (we also exclude countries with less than 10,000 resident users).

## Mobility profiles of countries

**Figure 3.**
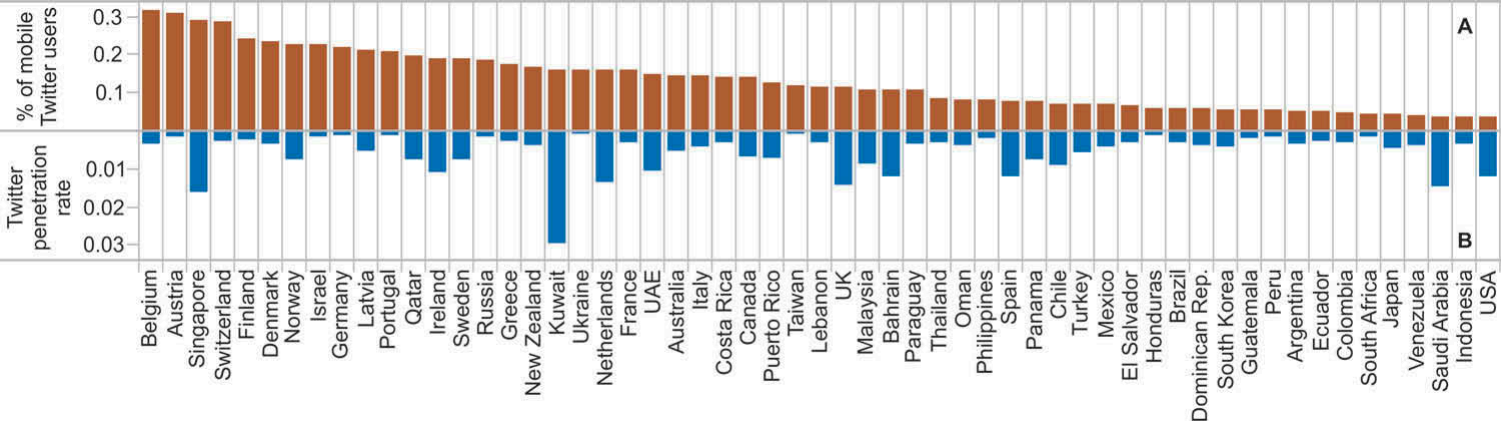
Countries with the highest rates of users’ travel activity.

Human mobility can be analyzed at different levels of granularity. In this study, we considered a user as being “mobile” if over the whole year the user had been tweeting from at least one country other than her or his country of residence. This applied to 1M users or around 8% of all those who used geo-located Twitter in 2012. Figure 3 shows the percentage of mobile users per country and the (geo-located) Twitter penetration in that country in 2012. Most of the top mobile countries, e.g., Belgium, Austria, were characterized by only moderate levels of Twitter adoption. On the other hand, users of geo-located Twitter from the USA, the country with the highest penetration rate, revealed a surprisingly small tendency to travel. The only two countries with high mobility and penetration rates were Singapore and Kuwait. In general, while an increased popularity of Twitter can be treated as a sign of a more active society, it did not immediately imply higher mobility of its users.

**Figure 4.**
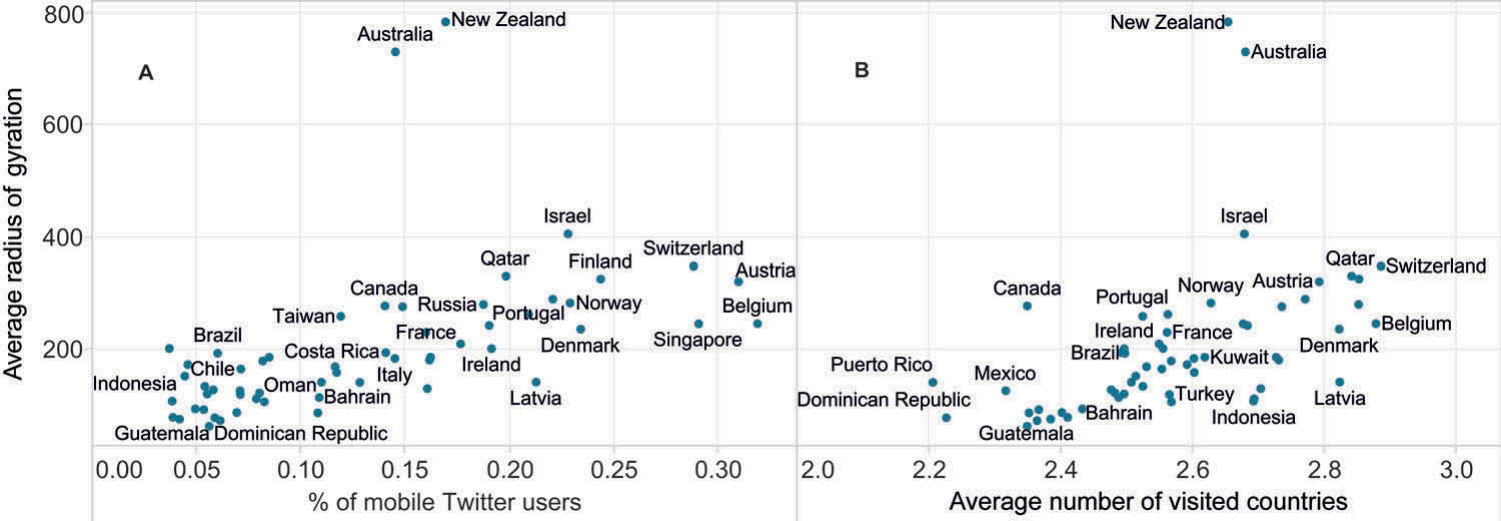
Average radius of gyration of users from different countries compared to (A) percentage of mobile Twitter users and (B) number of countries visited.

Next, we examined how spatially spread or concentrated the mobility of users is in a certain nation. This was captured through an average radius of gyration of the users. The radius of gyration measures the spread of user’s locations around her or his usual location. Here, we defined a usual location as the center of mass rather than a home location, as the latter was defined too broadly, at the level of a particular country. For each user, the radius of gyration was computed thus:

(1) 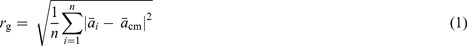

where *n* is the number of tweeting locations, *ā*_i_ represents the location of a particular tweet (a pair of *x–y* coordinates), and ā_cm_ is a user’s center of mass. Low values of the radius indicate a tendency to travel locally, while higher values indicate more long-distance travels. The average values computed for users from different countries are shown in Figure 4. At first glance, it is obvious that the geographical location of a country played an important role. Isolated countries such as New Zealand or Australia had an average radius of gyration of over 700 km. There was also a positive correlation between the average distance travelled by residents of a country and the mobility rate of its Twitter population, as well as the number of visited countries (Figure 4A and B). This notwithstanding, the drivers for increased mobility were invariably linked to the economic prosperity of a country, as all received rankings were led by highly developed countries.

**Figure 5.**
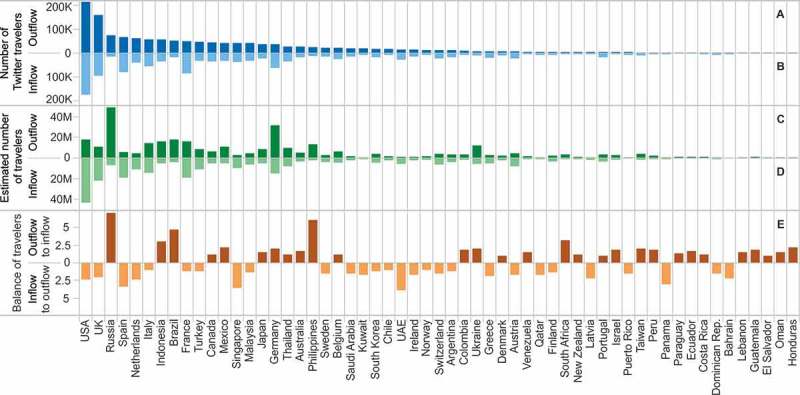
Number of visitors coming from or arriving in a country. (A and B) Number of Twitter travelers, (C and D) estimated total number of travelers (number of Twitter travelers normalized by the Twitter penetration rate in the country of origin of the visitor), and (E) the yearly ratio between the estimated inflow and outflow of travelers.

The mobility profile of each country can be analyzed from two perspectives, as being either the origin or the destination of international travel. By building the directional country-to-country network of human travels, we were able to quantify both the inflow and outflow of visitors. Figure 5 shows the results of country-specific analyses based on Twitter users and on the estimated total number of travelers. Figure 5A shows the number of Twitter users residing in a country and traveling to another and Figure 5B shows the number of users visiting this particular country. Figure 5C and D present the number of Twitter travelers normalized by the Twitter penetration rate in the user’s home country. In the case of inflow of travelers, both the raw number of Twitter users and the estimated population of visitors point to USA, UK, Spain, and France to be the most visited countries. The nationality of outgoing travelers seemed highly influenced by Twitter’s penetration rate, with the biggest groups coming from countries of high Twitter popularity. Low penetration indices lead to an overestimation of the actual volume of travel, which may explain the high values estimated for Russia or Germany. Figure 5E presents the yearly ratio between the estimated inflow and outflow of travelers, revealing which countries were the origin or destination of international travel.

## Temporal patterns of mobility

**Figure 6.**
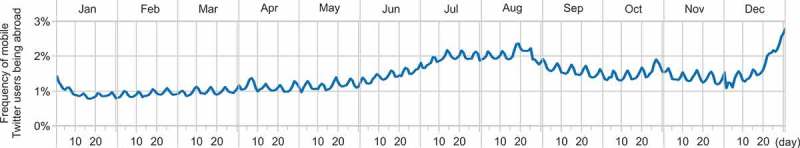
Global temporal pattern of abroad travels by Twitter users.

Human mobility is subject to temporal variations. In order to uncover patterns occurring at the global level, as well as at the country level, we measured how many Twitter users were active outside of their country of residence for each day of 2012. The first pattern that emerged from the analysis was the weekly scheme of check-ins abroad (Figure 6). The tendency of increased mobility over weekends seemed to be universal across the globe. Moreover, there were two obvious seasons of higher mobility: the summer months of July and August and the end of the year, connected to Christmas and New Year’s Eve holidays.

**Figure 7.**
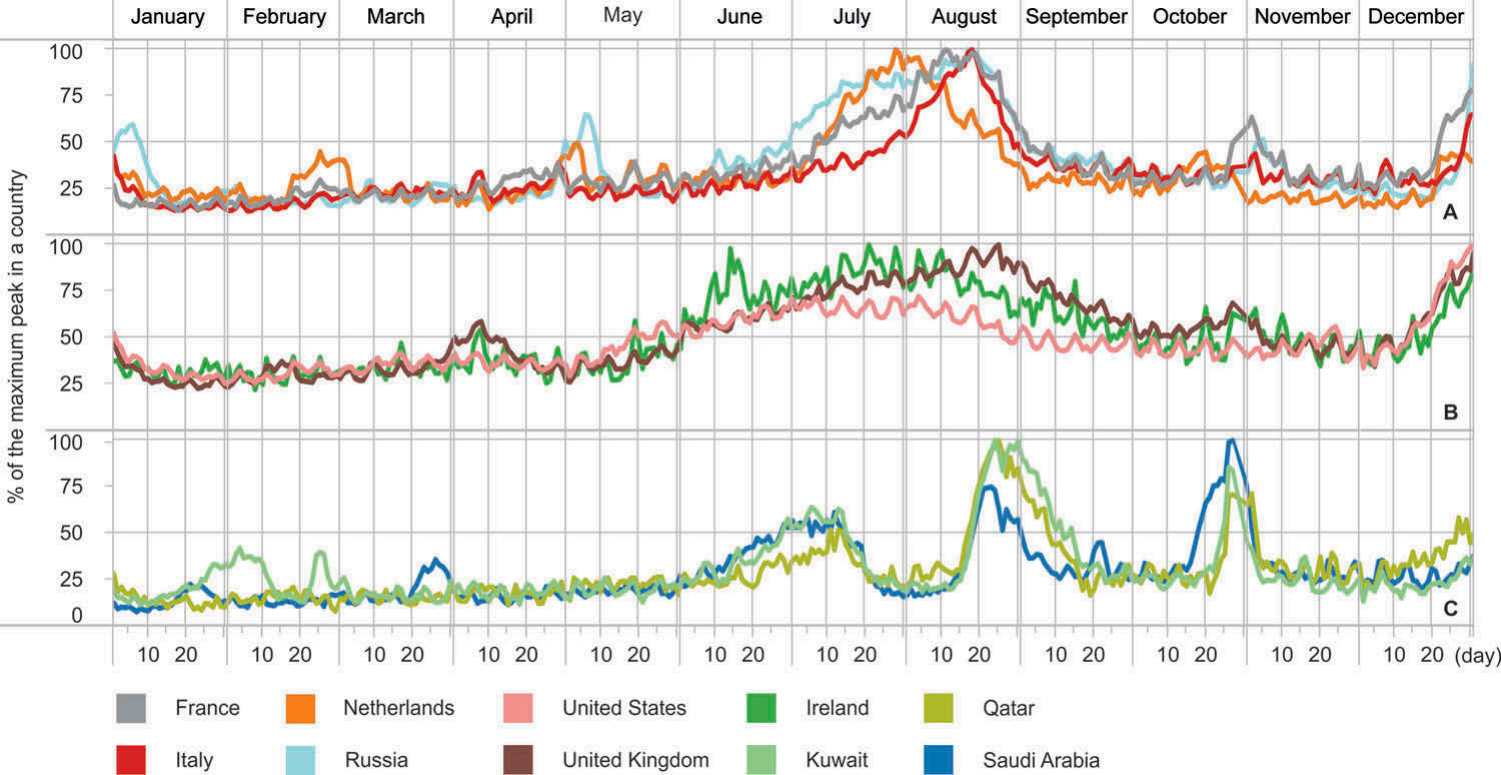
Normalized temporal patterns of mobility, by country of origin. The values for each country are scaled between 0 and 100% of the maximum daily number of travelers being abroad during 2012.

When specific countries were considered, we discovered a variety of deviations from the aforementioned global pattern. Several of them were easy to interpret and were shared by more than one country. For instance, there was a substantial group of European nations with the biggest peak over one of the summer months and a few smaller ones, most probably connected to extended weekends, e.g., at the beginning of May (examples are shown in Figure 7A). Another group exhibited a similar pattern; however, the summer mobility increase for this group stretched between June and September (Figure 7B). An interesting example of how the mobility behavior is influenced by the social and cultural norms of a country was observed in a group of Arabic countries (Figure 7C). The period of Ramadan corresponded to a major decrease in the amount of travel abroad, while the time of the Mecca pilgrimage at the end of October was marked by a sharp peak. In all cases, increased international mobility corresponded with the end of the year.

**Figure 8.**
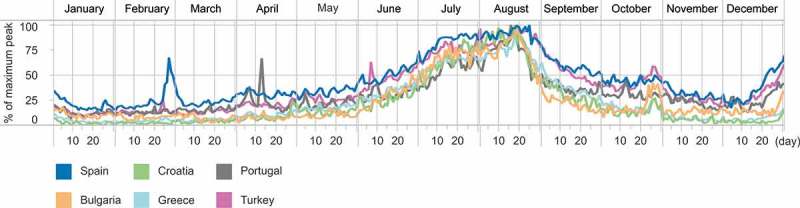
Destinations of tourist activity with increased inflow of international Twitter users over summer. The values for each country are scaled between 0 and 100% of the maximum daily number of international visitors during 2012.

The temporal variations of the inflow of visitors were much more stable than those of the outflow patterns. Visual inspection of those patterns identified three main groups. The first group included countries without any specific seasonality of international arrivals (with the exception of the end of the year). The second group covered popular summer destinations such as Spain, Italy, Croatia, or Greece (Figure 8), with a significant increase in arrivals over the months of July and August. Finally, in the third group, we included countries where increased international arrivals were connected to special events such as Euro 2012 in Poland or the 2012 Olympics in the UK.

## Country-to-country network and partitioning

Next, we analyzed the topology of the country-to-country mobility network created by travelers within the Twitter community. As it has already been proven by many other studies, partitioning of a raw network of human communication interactions, e.g., based on mobile phone data (Blondel 2011; Blondel, Krings, and Thomas 2010; Ratti et al. 2010; Sobolevsky, Campari, et al. 2013; Sobolevsky, Szell, et al. 2013), as well as partitioning of human mobility (Amini et al. 2013; Kang et al. 2013), can lead to the delineation of spatially cohesive communities, aligning surprisingly well with the existing socioeconomic borders of the underlying geographies. Our aim was to test if this finding holds true for the Twitter-based mobility network, and if so, which distinctive mobility clusters emerge in different parts of the world.

**Figure 9.**
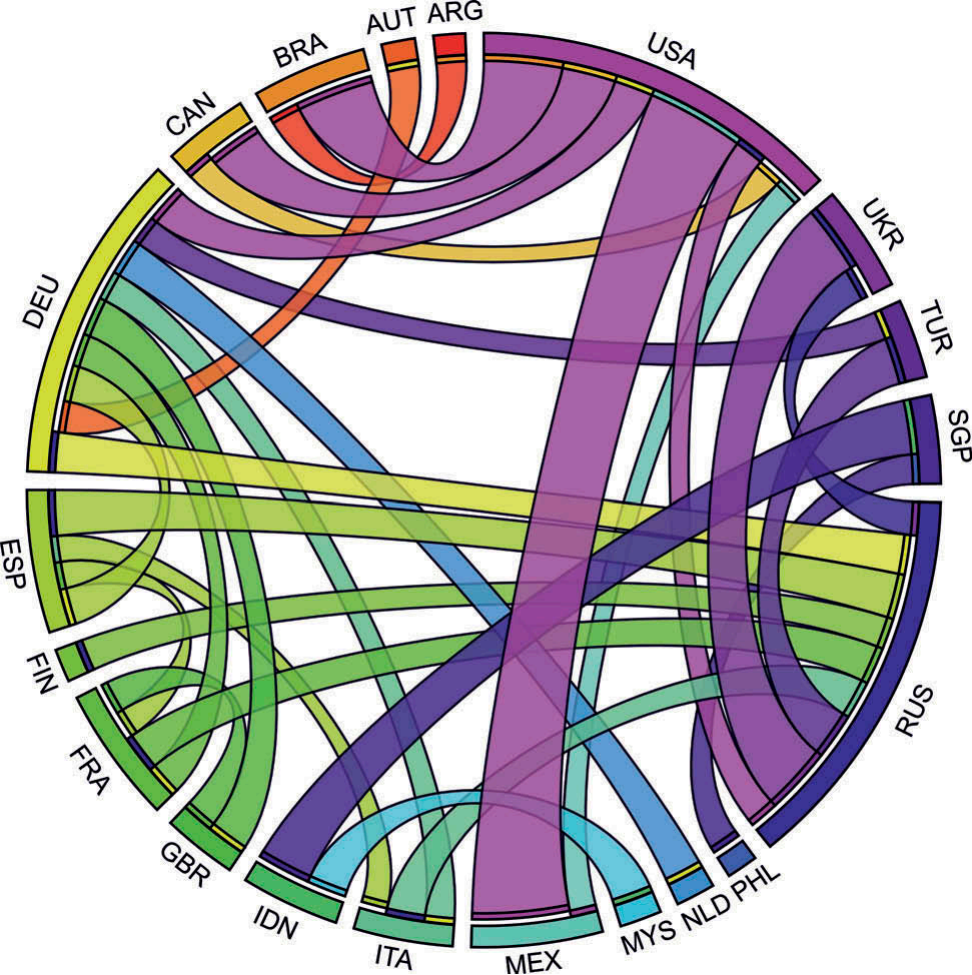
Top 30 country-to-country estimated flows of visitors. Colors of the ribbons correspond to the destination of a trip; the country of origin is marked with a thin stripe at the end of a ribbon (visualization method based on Krzywinski et al. 2009).

Taking advantage of our methodology of assigning users to their country of residence and focusing on mobile users, we built a worldwide country-to-country network of tweet flows. Each country was considered as a node, and the edges of the network were weighted with the number of Twitter users traveling between a pair of nodes. The network was directional, as the connections were built from the country of residence to countries where a user appeared as a visitor. To deal with the sparseness of the network and different levels of Twitter representativeness, we filtered out all countries with the outgoing Twitter population smaller than 500 users and countries where the Twitter penetration was below 0.05%. The flows were normalized by the Twitter penetration rate in the country of a user’s origin in order to estimate the real mobility flux rather than just a number of Twitter users. The top 30 flows between different countries are presented in Figure 9.

The network partitioning procedure was based on the well-known modularity optimization approach (Newman 2006) and uses a highly efficient optimization algorithm recently proposed by Sobolevsky, Campari, et al. (2013). The procedure assesses the relative strength of particular links versus the estimations of the homogenous null model. It optimizes the overall modularity score of a network partitioning, which quantifies the strength of intracluster connections (in the “ideal” partitioning case they should be as strong as possible) and the weakness of outer ties (should be as weak as possible).

**Figure 10.**
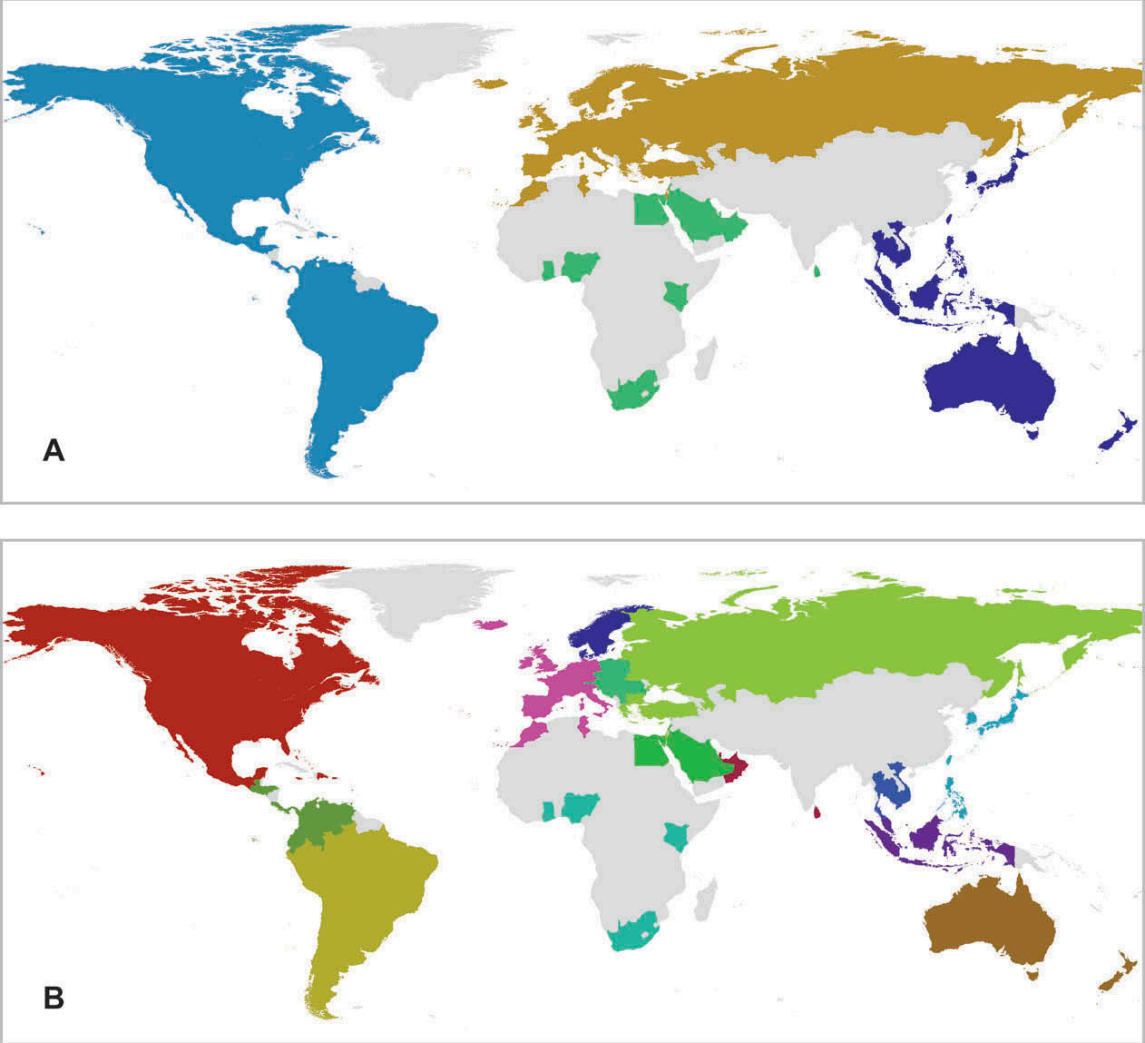
Mobility regions uncovered by the partitioning of the country-to-country network of Twitter user flows. Regions distinguished at the first (A) and second (B) level of partitioning. Gray color indicates no data.

After obtaining the initial split of the network, the partitioning procedure was applied iteratively to the subnetworks inside each community, much as Sobolevsky, Szell, et al. (2013) did. As a result, the Twitter network was split into mobility clusters on three hierarchical levels, each level being a subpartitioning of the previous one. The initial level (Figure 10A) uncovered four groups of countries that reflected the continental division of the world. In this sense, travel connections between North and South America were stronger than that between America and Europe, while the Europeans traveled more within Europe and to Asia than to the other continents. Further subdivisions followed the same type of logic, the clusters tending to be spatially connected and well aligned with common socio-geographical regions. For instance, on level 2, we observed a split into Western, Central, Eastern, and Northern Europe (Figure 10B) and on level 3, Central Europe was further divided into the more continental northern part and the Balkans (Table 1). Received mobility clusters intimate that people tend to travel more to close-by destinations rather than further afield. Furthermore, the finding that clusters were spatially continuous and reflected common world regions is in line with the findings of previous studies with mobile phone data (Blondel 2011; Blondel, Krings, and Thomas 2010; Ratti et al. 2010; Sobolevsky, Szell, et al. 2013). The finding also extends the validity of network-based community detection from a country to global scale. Additionally, we see that the partitioning of mobility networks follows the same as human communication networks and might thus be used for regional delineation purposes.

**Table 1.** Countries assigned to different regions of mobility.

Level 1	Level 2	Level 3	Assigned countries
1	1	1	Bahamas, Canada, Dominican Republic, Jamaica, Mexico, Puerto Rico, USA
	2	2	Colombia, Ecuador, Panama, Trinidad and Tobago, Venezuela
		3	Costa Rica, El Salvador, Guatemala, Honduras
	3	4	Bolivia, Chile, Peru
		5	Argentina, Brazil, Paraguay, Uruguay
2	4	6	France, Ireland, Malta, Martinique, Morocco, Portugal, Spain, Tunisia, UK
		7	Belgium, Germany, Iceland, Italy, Luxembourg, Netherlands, Switzerland
	5	8	Denmark, Norway, Sweden
	6	9	Austria, Czech Republic, Hungary, Poland, Romania, Slovakia
		10	Bosnia and Herzegovina, Croatia, Kosovo, Macedonia, Serbia, Slovenia
	7	11	Azerbaijan, Bulgaria, Cyprus, Greece, Israel, Kazakhstan, Latvia, Lithuania, Russia, Ukraine
		12	Belarus, Estonia, Finland, Turkey
3	8	13	Ghana, Nigeria
		14	Kenya, South Africa
	9	15	Bahrain, Egypt, Jordan, Kuwait, Lebanon, Saudi Arabia
	10	16	Oman, Qatar, United Arab Emirates
		17	Maldives, Sri Lanka
4	11	18	Japan, South Korea, Taiwan
		19	Philippines
	12	20	Brunei, Indonesia, Malaysia, Singapore
	13	21	Cambodia, Thailand, Vietnam
	14	22	Australia, New Zealand

## Validation of the results

**Figure 11.**
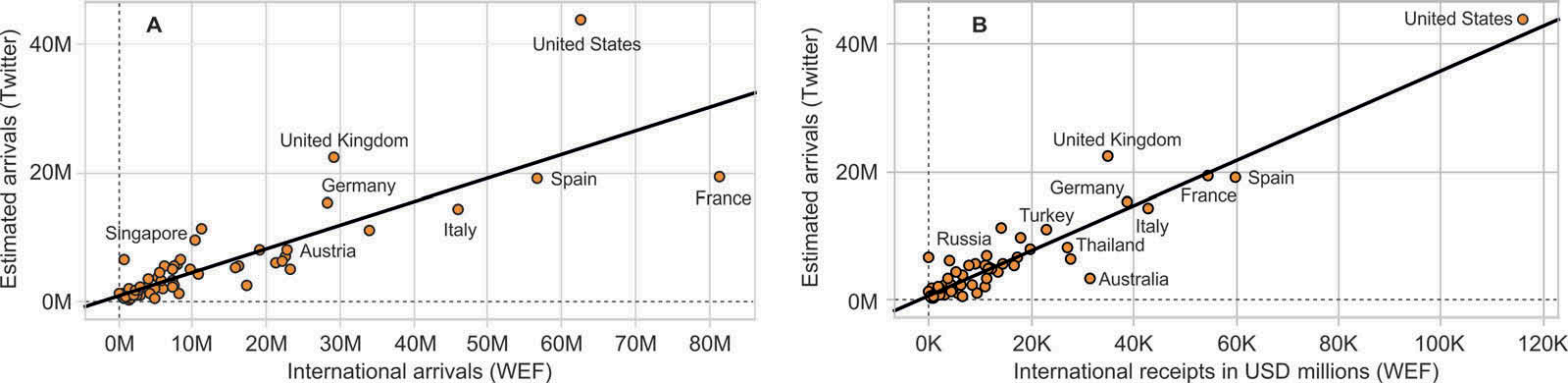
International arrivals estimated with Twitter data versus the arrivals (A) and nominal value of tourist receipts (expenditures by international inbound visitors, B) provided by WEF (2013). *R* ^2^ statistic equals 0.69 and 0.88, respectively.

For global mobility, it is difficult to find a bias-free human mobility dataset that would enable direct validation of results obtained with Twitter. An interesting comparison could be made, for instance, with the register of flight connections, although this could be hampered by the possibility of confounding a segment of an indirect travel for direct travel from one’s home country to an intended destination. In practice, such a comparison is also obviously prevented by the difficulty of accessing such data. In this study, we relied on tourism statistics provided by the World Economic Forum (WEF 2013) at the country level. We used two of those statistics: international tourist arrivals (thousands, 2011) and international tourism receipts (US$, millions, 2011) and compared them to arrivals estimated on the basis of the Twitter data (Figures 11A and 10B). In both cases, we found a strong linear correlation (respectively with the *R* ^2^ of 0.69 and 0.88), which confirms the validity of the estimated mobility figures.

**Figure 12.**
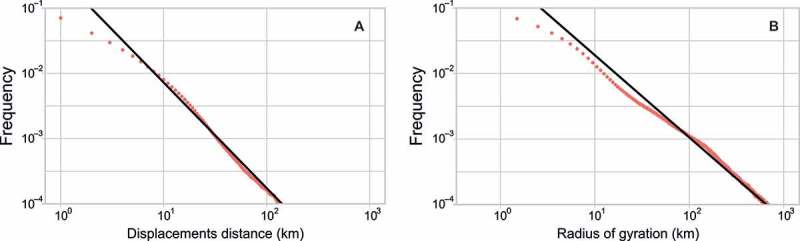
Probability of displacement (A) and frequency of the radius of gyration (B).

We further validated the results indirectly by demonstrating that mobility measures derived from Twitter activity exhibit similar statistical properties as those obtained using other datasets. First, we computed the distance between each pair of consecutive user locations (tweets) and plotted the frequencies of computed displacement on a log-log scale. Similarly to other studies, we found that the distribution is well approximated by the power law (Figure 12A):

(2) 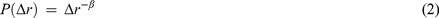

where Δ*r* is a displacement of certain length and *β* = 1.62.

Importantly, the received exponent stays in the similar range as the exponents obtained with other mobility datasets such as mobile phone data (González, Hidalgo, and Barabási 2008, *β* = 1.75), bank note dispersal (Brockmann, Hufnagel, and Geisel 2006, *β* = 1.59), and Foursquare check-ins (Cheng et al. 2011, *β* = 1.88). We also plotted the frequency of previously computed users’ radiuses of gyration (Figure 12B). As expected, it also followed a power law with an exponent of 1.25.

**Figure 13.**
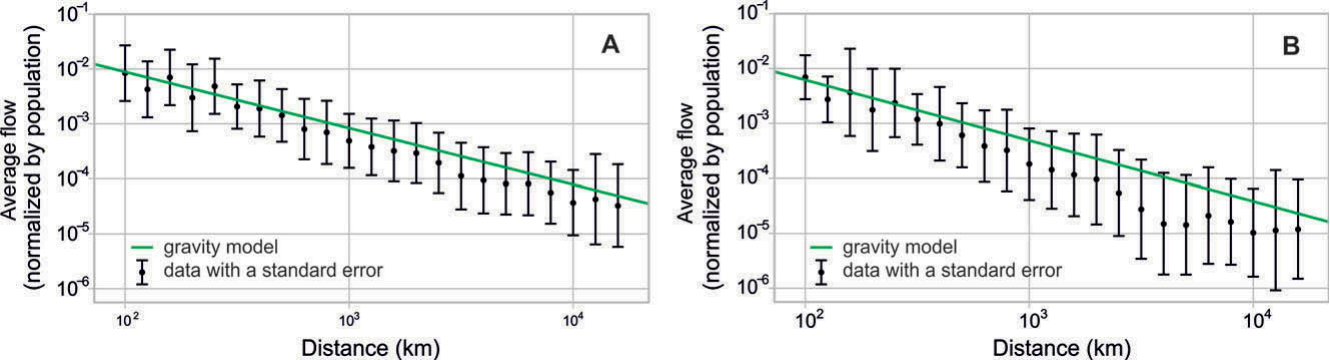
Dependence of human flow (*F_ij_*) normalized with populations in countries of origin and destination (*A p_i_^α^p_j_^β^*) on the distance in comparison to a distance decay function (*r^–γ^*) modeled with the gravity law. (A) Network defined based on raw Twitter flows. (B) Network of total population flows estimated with the Twitter penetration rate in the country of origin.

Given the limited access to global mobility data suitable for a direct comparison with Twitter-based human travels, we further tested our data against a commonly accepted mobility model – the classic gravity approach – as yet another way of indirect verification of uncovered patterns. Many studies proved the gravity law to provide a good basis for modeling the intensity of interactions between locations, depending on their weights and distance, in the context of not only mobility (e.g., Balcan et al. 2009; Jung, Wang, and Stanley 2008; Zipf 1946) but also human interaction networks (Expert et al. 2011; Krings et al. 2009). We made the assumption that if the Twitter data were to be considered suitable for a description of human mobility, they should follow a similar law and exhibit similar distance dependence as those found for railway connections, airline traffic, mobile telecom records, and other data. We also tested how much the gravity law holds on a global scale, especially in times when the influence of distance as a barrier to mobility is often considered to decrease in importance. We used the gravity model in the form:

(3) 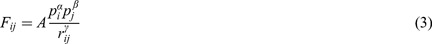

where *F_ij_* represents a flow of people between a pair of countries, *p_i_* is the population in the country of origin, *p_j_* the population of the destination country, *r_ij_* is the distance measured between the capitals, and *A* is a constant. To compensate for the limitation of our definition of country-to-country distance, we restricted the connections to the countries that are at least 100 km apart. The model was fitted to the two versions of our network. First, the flows were defined as a raw number of Twitter users traveling between two countries, and the population of each country was equal to the number of Twitter residents (Figure 13A). The exponents were *α* = 0.81, *β* = 0.63, and *γ* = 1.02 and the *R* ^2^ coefficient was 0.79. The second variation of the model (Figure 13B) used flows estimated based on the Twitter penetration rate (again with the threshold of 0.05%) and population provided by the Central Intelligence Agency (2012). The exponents found were fairly similar to the previously found exponents – *α* = 0.89, *β* = 0.69, and *γ* = 1.1 – but the *R* ^2^ coefficient (0.71) was slightly lower.

The population exponents we obtained suggest that a country’s size influences the growth of human flow sublinearly, in terms of both origin and destination of travel, but the influence of the population in a country of origin is bigger. This relationship can be explained with two conjectures. On the one hand, it is plausible that residents of a country do not take part in mobility in an equal manner; rather it is a domain of the most active residents. On the other hand, it could be that visitors were never attracted by the whole country but only by certain places within the country. The number of active users and attractive places in countries may on average grow slower than the countries’ total population.

The gamma exponents suggest, as expected, a decrease of interaction intensity with distance (Figure 13A and B), however at a slower rate than often pre-assumed *r* ^2^ decay relationship, e.g., by Jung, Wang, and Stanley (2008) or Krings et al. (2009). The difference in received decay relationship can be explained by the scale of analysis. On a global scale, where most of the trips are happening by air, an increase in distance comes with relatively smaller effort or cost than on a country or local levels where most travel is by land. But even in the world of this subjective “shrinkage” of distances, certain level of dependency is preserved, possibly because of social ties, which remain stronger on a local than global scale (Takhteyev, Gruzd, and Wellman 2012). In other words, people may simply have more reasons to travel shorter distances, which also correlates well with the results received during network partitioning.

Visually, both models seem to be well fitted (Figure 13A and B), with the slopes reflecting well the average tendency in the observed data and the distant decay functions remaining within the standard errors across the distance range. The similarity of both models suggests that Twitter data not only may provide a valid picture of mobility of its direct users, but can further be used for the estimation of real human flows.

## Conclusions

Geo-located Twitter is one of the first free and easily available global data sources that store millions of objective, digital records of human activity in space and time. In our study, we demonstrated that, despite the unequal distribution over the different parts of the world and possible bias toward a certain part of the population, in many cases geo-located Twitter can and should be considered as a valuable proxy for human mobility, especially at the level of country-to-country flows. Our approach proposed to capture nation-specific mobility by assigning users to their country of residence. As a result, we were able to compare mobility profiles of countries, considering each country as both a potential origin and a destination of international travels. The results showed that increased mobility (measured in terms of the probability of travel, diversity of destinations, and geographical spread of travels) is characteristic of West European and other developed countries. Traveling distance was additionally affected by the geographic isolation of a country, such as Australia or New Zealand. Through the analysis of temporal patterns, we found a globally universal season of increased mobility at the end of the year. Although the summer mobility was increased for a wide range of countries, it varied in terms of intensity and duration, and in some cases there was no increase at all. Additionally, we discovered patterns driven either by cultural conditioning or by special events occurring in a country. In many cases, the results well confirmed logical expectations, which we treat as an indicator of the legitimacy of Twitter as a global and objective register of human mobility.

Furthermore, we demonstrated that the communities detected using a Twitter mobility network formed spatially cohesive regions reflecting the regional division of the world. This finding is important from several standpoints. First, it is in agreement with the results obtained by Blondel, Krings, and Thomas (2010), Ratti et al. (2010), and Sobolevsky, Szell, et al. (2013) who based their research on networks of mobile phone interactions. Second, it expands the spatial validity of the community detection approach from a previously examined country scale to the global scale. Third, it shows that even in the era of globalization and seeming decrease of the influence of distance, people still tend to travel locally, visiting neighboring countries more often than those further away.

Further, we validated, to a certain extent, geo-located Twitter as a proxy of global mobility behavior. We demonstrated that the number of visitors estimated for different countries based on Twitter data is in line with the official statistics on international tourism. The correlation (*R* ^2^ around 0.7) shows a fairly good correspondence, given the wider scope of mobility captured through Twitter and a significantly different method of data acquisition. Further, we confirmed that Twitter data exhibit similar statistical properties as other mobility datasets. For instance, measures such as radius of gyration and probability of displacement are well estimated with the power law distribution (similarly as in Cheng et al. 2011; Brockmann, Hufnagel, and Geisel 2006), while the network of estimated flows of travelers can be described fairly accurately using the classic model of a mobility – the gravity model.

We believe the analysis presented in this article proves the potential of geo-located Twitter as an objective, freely accessible source of data for global mobility studies. Further research will focus on exploring the applicability of Twitter activity for finer spatial scales.

## Notes

1. By geo-located, we mean the messages with explicit geographic coordinates attached to each message.

2. 
https://dev.twitter.com/docs/streaming-apis
